# High-resolution observations on enrichment processes in the sea-surface microlayer

**DOI:** 10.1038/s41598-018-31465-8

**Published:** 2018-09-03

**Authors:** Nur Ili Hamizah Mustaffa, Thomas H. Badewien, Mariana Ribas-Ribas, Oliver Wurl

**Affiliations:** Institute for Chemistry and Biology of the Marine Environment, Carl Von Ossietzky Universität Oldenburg, 26382 Wilhelmshaven, Germany

## Abstract

For decades, researchers assumed that enrichment of dissolved organic matter (DOM) in the sea surface microlayer (SML) is solely controlled by changes in the DOM concentration at this uppermost thin boundary layer between the ocean and the atmosphere. We conducted high-resolution observations of fluorescent-DOM (FDOM) at 13 stations in the coastal and open Atlantic Ocean to understand the enrichment processes. Results show that FDOM enrichment in the SML varied between 0.8 and 2.0 (in comparison to the concentrations in the underlying water; ULW), and FDOM enrichment is a common feature of the SML despite the varied distances to the terrestrial sources. At six stations, the FDOM concentration in the SML was less variable over the sampling period (>5 h) compared to FDOM concentrations in the ULW characterized with sudden changes. Even so we observed slightly lower enrichments with increasing wind speeds and solar radiation, changes in ULW concentrations forced the enrichment to change. In addition, we found evidences for the occurrence of photochemical degradation of FDOM in near-shore SML with implications on coastal carbon cycling. Overall, the results show that the processes leading to the enrichment of DOM in the SML are more complex than previously assumed. Given the importance of the organic-rich SML as a diffusion layer in the air–sea exchange of climate-relevant gases and heat, understanding the layer’s enrichment processes is crucial.

## Introduction

The sea surface microlayer (SML) is defined as the uppermost boundary layer of the ocean and is enriched with a complex pool of surface-active substances, including carbohydrates, proteins, and lipids^1^. Despite a thickness ranging from tens of microns to 1 mm depending on the sea state, the ubiquitous coverage of the ocean with the SML^2^ is known to be important for biogeochemical and climate-relevant processes on a global scale^3^. For example, the enrichment of surface-active substances creates a laminar layer that reduces the air–sea gas exchange rates^4–6^ and therefore, has a direct impact on the fate of climate-relevant gases in the atmosphere. Additionally, a wide range of microbes^7^ colonizes the enriched organic matrix in the SML turning the SML into a biofilm-like habitat under certain conditions^8^. Bursting of ascending bubble plumes after wave breaking^9^ transfers organic materials and bacteria from the SML to aerosols, and researchers have shown such material on aerosols influences the behavior of ice-nucleation and cloud formation^10,11^ with a negative feedback on climate change. Furthermore, the organic matrix of the SML traps human-made toxic chemicals and pathogens^12^, which affects the complete food web from fish larvae^13^ to surface-dwelling top predators^14^. The effects of the SML on climate change and the marine food web are based on the enrichment of organic material in the SML. For this reason, it is crucial to understand the processes leading to enrichment, which is defined as ratios of concentrations or abundances between the SML and the underlying water (ULW). Hardy^15^ reviewed processes leading to the enrichment of organic material in the SML, including upwelling, convection, diffusion, and rising bubble plumes from the underlying water column. Furthermore, photosynthetic plankton in the SML exudes higher amounts of organic molecules due to stress conditions under high levels of radiation^16^. Wet and dry^17^ deposition of atmospheric aerosols leads to the enrichment of particulate material in the SML compared to the ULW. All these processes lead to a change in the concentrations in the SML, rather than in the ULW, and, therefore, researchers have assumed that mainly changes in the concentrations or abundances in the thin SML force enrichment. In addition, understanding enrichment processes are crucial for remote sensing data^18,19^ as satellite’s data retrieval occurs in the thin ocean’s surface with an exponential decay of signals to a depth of 1 cm^20^. The aim of this study is to answer the general question whether the magnitude of enrichment of dissolved organic matter (DOM) is solely forced by changes in concentrations in the SML, or to what extent and which processes in the ULW may be involved. For this purpose, we made observations from a state-of-the-art research catamaran and high-resolution *in situ* measurements of the fluorescent dissolved organic matter (FDOM), temperature, and salinity in the SML and the ULW, as well as meteorological parameters.

## Results

### FDOM enrichment at different water masses

**Figure 1:**
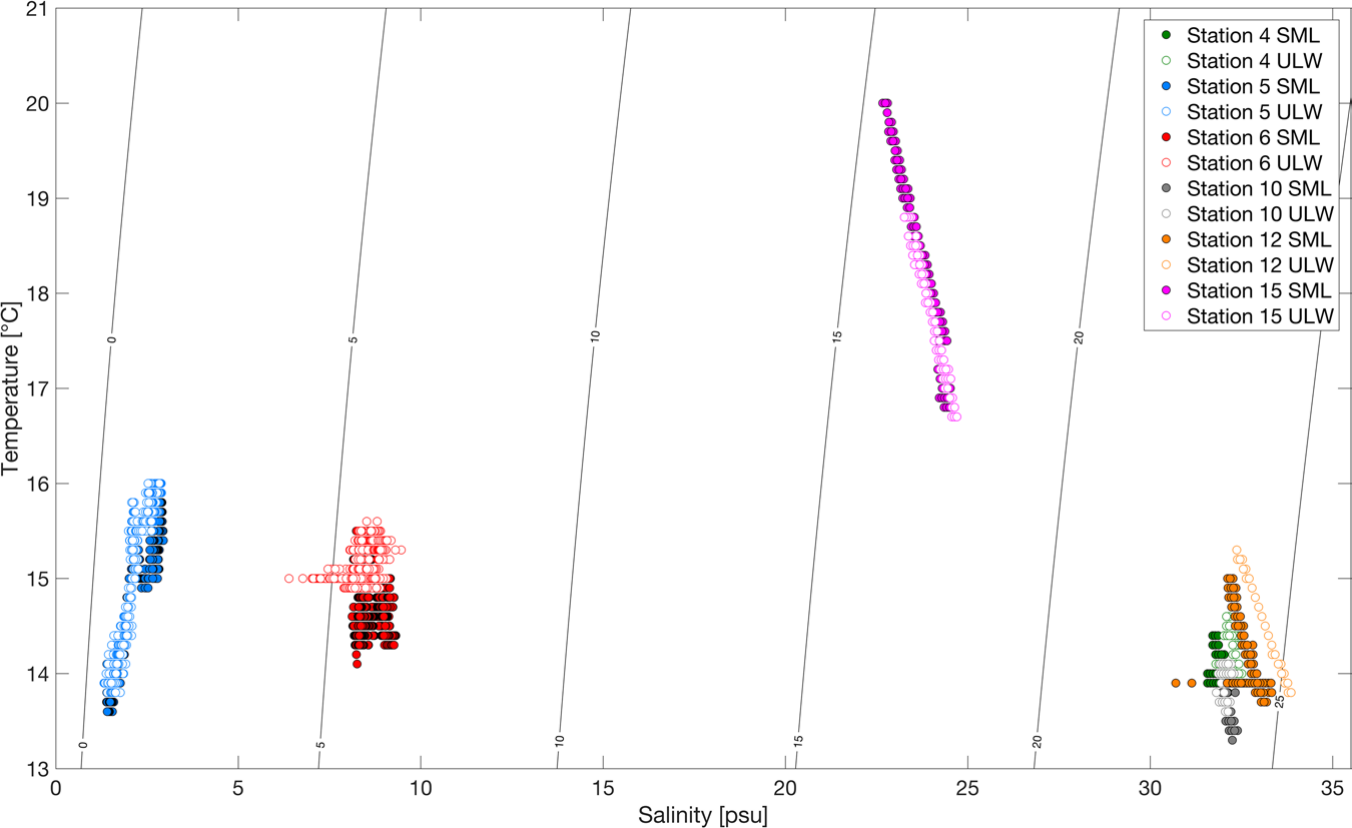
Temperature-salinity (T-S) diagram at the six selected stations showing the distribution of different water masses. The contours represent lines of equal density, expressed as σ_*t*_ (Sigma-t). SML: sea surface microlayer, ULW: underlying water.

**Figure 2:**
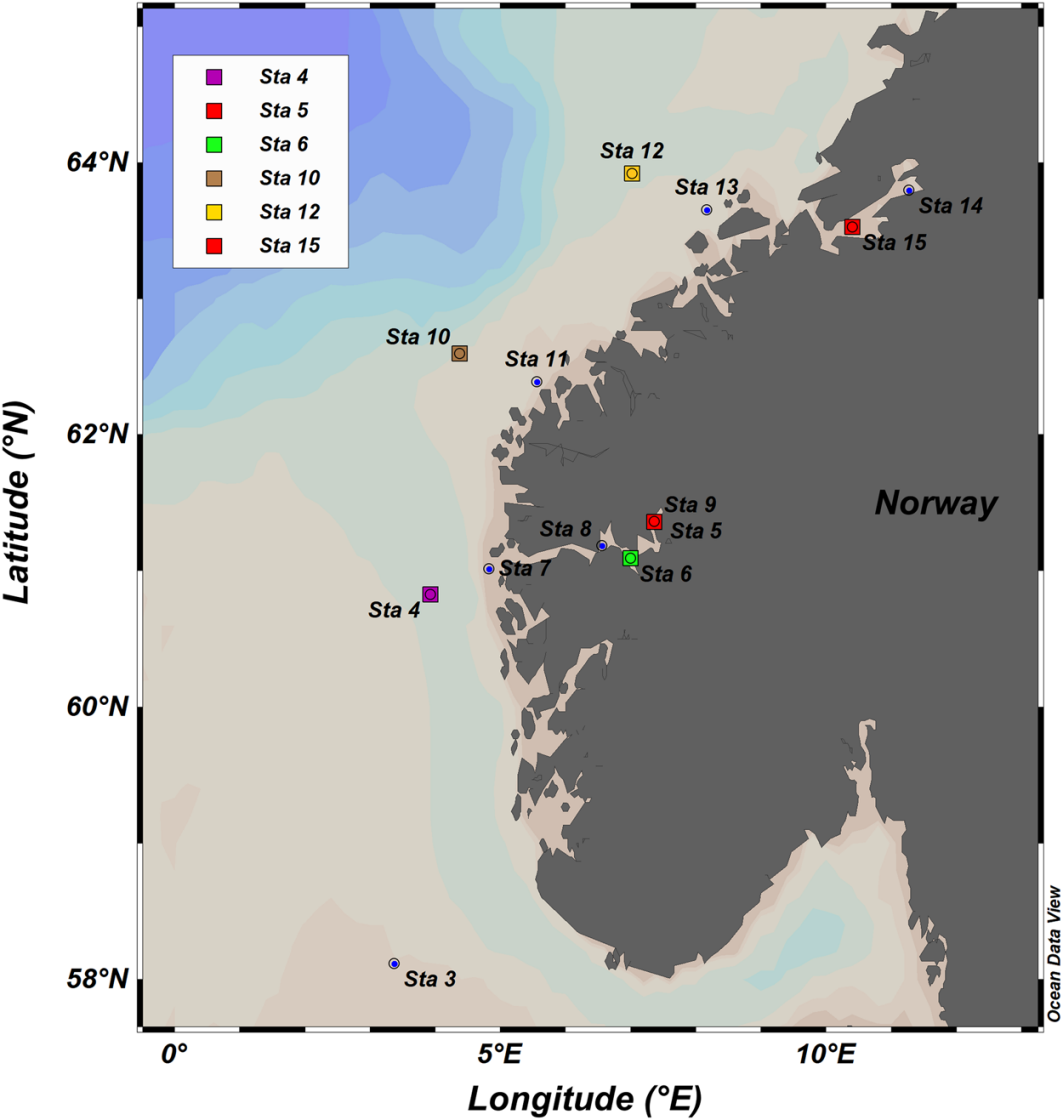
Map of the sampling stations during the HE491 cruise. The six selected stations are marked accordingly. The map was plotted using Ocean Data View (ODV) Version 4.4.10^45^.

The temperature-salinity (T-S) diagram (Fig. 1) shows the different water masses observed at the six stations (Fig. 2). We selected six out of 13 stations for detailed analysis based on the observed water masses influencing the enrichment factor of FDOM in the SML, and we provide the details in Table S1. Overall, the T-S diagram indicates distinct clusters of data points for SML and ULW, indicating that the SML diverts from temperature and salinity features of the ULW. Despite distinct temperature and salinity features, the T-S diagram shows for Stations 5 and 6 shows generally similar densities in the SML and the ULW, suggesting the presence of small-scale and local mixing at the sea surface. Meanwhile, densities for Stations 4, 10, 12 and 15 changes over the sampling period with a linear trend. Clearly noticeable for Station 12 is the low-density SML being well stratified from the ULW, with similar but less pronounced observations at Station 4 and 15.

The results for the FDOM concentrations, enrichment factors, photosynthetic yields, and other physical parameters are listed in Table S1. Overall, the FDOM concentration in the SML ranged between 2.0 and 17.6 µg L^−1^ (6.1 ± 0.4 µg L^−1^, *n* = 13642) with the highest concentration (15.7 ± 1.0 µg L^−1^, *n* = 2507) found in the Trondheim Fjord (Station 15, Table S1). The FDOM concentration in the ULW varied between 2.8 and 18.8 µg L^−1^ (6.0 ± 0.3 µg L^−1^, *n* = 13642). The enrichment factor of the FDOM ranged between 0.8 and 2.0 (average: 1.1 ± 0.1, *n* = 13642). A whisker box plot (Fig. S1) shows that 75% of the observations at Stations 4, 5, 6, 10, and 12 show enrichment (enrichment factor >1) of the FDOM in the SML with an average value of 1.2 ± 0.1 (*n* = 11135). We observed continuous depletion (enrichment factor <1) at Station 15 (average: 0.9 ± 0.1, *n* = 2507). Analysis of variance (Kruskal Wallis test, *p* < 0.001) with post-comparison test (Dunn test) revealed that Station 12 (located in the North Atlantic Ocean), had a considerably higher enrichment factor (average = 1.3 ± 0.2, median = 1.3, *n* = 2489) compared to other stations, although Station 12 is located 93 nautical miles offshore from the Trondheim Fjord, e.g., from terrestrial sources (Fig. 2). The average quantum yields at Station 12 (the highest enrichment factor) and Station 15 (the lowest enrichment factor) were 0.4 ± 0.2 (*n* = 1990) and 0.3 ± 0.1 (*n* = 2507), respectively. The medians of the enrichment factors in the inner (Station 5) and middle (Station 6) parts of the Sogne Fjord were 1.1 and 1.2, respectively, and the difference was not statistically significant (Mann-Whitney test, *p* = 0.2047). The medians of the enrichment factors at Stations 4 and 10 (Atlantic Ocean) were 1.1 and 1.2, respectively, and within the same range as in the Sogne Fjord despite the different distances of the potential terrestrial FDOM sources. The FDOM concentrations in the Sogne Fjord were in a similar range as the concentrations we found in the Atlantic Ocean but were three to eight times lower compared to those in the Trondheim Fjord. This result shows that the Sogne Fjord is not an important source of terrestrial FDOM for the Atlantic Ocean, probably due to the fjord’s different topography features^21^, e.g., the shallow sill at the main entrance separating the deep water basin from the adjacent coastal water.

**Figure 3:**
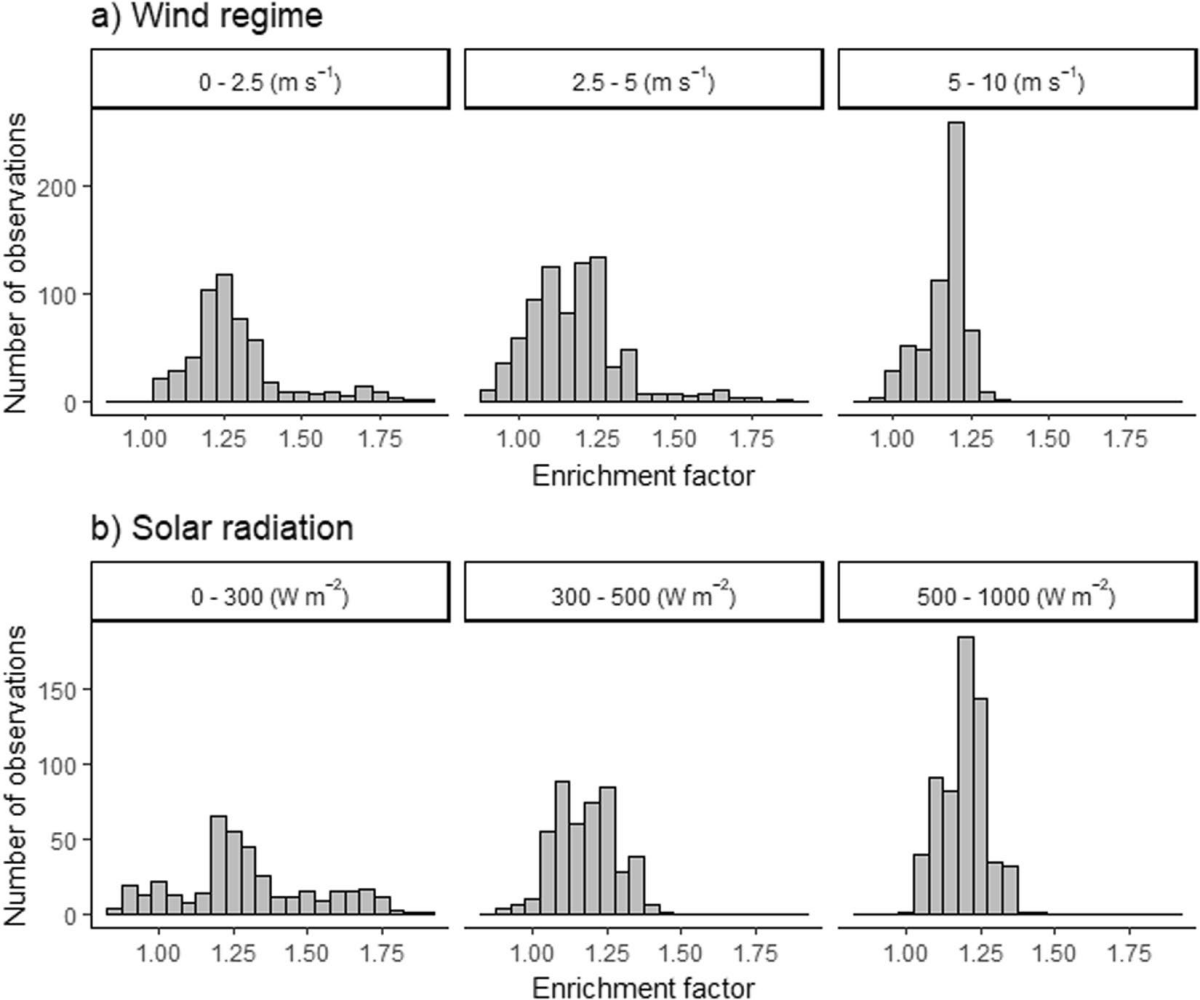
Histograms for observed enrichment factors at three categorical levels for (**a**) wind speed and (**b**) solar radiation.

Figure 3 shows the histograms of enrichment factors for all stations (except Station 15) separating into three wind speed and solar radiation categories. Station 15 was excluded due to the immediate proximity to the shoreline with fresh inputs of terrestrial FDOM discussed further below. Categories for wind speed (0–2.5 m s^−1^, 2.5–5 m s^−1^ and 5–10 m s^−1^) and solar radiation (0–300 W m^−2^, 300–500 W m^−2^ and 500–1000 W m^−2^) are based on the Pierson Moskowitz sea spectrum^22^, and our observation on solar radiation during dawn, overcast and overall clear skies, respectively. The maximum numbers of observations on enrichments are similar for all three wind and radiation categories, but observed over a narrower range for the highest wind regime (5–10 m s^−1^) (Fig. 3a). The enrichment at the highest wind regime (5–10 m s^−1^) were statistically significant (*p* < 0.01) lower compared to enrichments for the low and moderate regimes (Kruskal Wallis with Dunn post-comparison test, *p* < 0.01). Similarly, enrichments at the high radiation regime (500–1000 W m^−2^, Fig. 3b) were significant lower compared to the low and moderate regimes (Kruskal Wallis with Dunn post-comparison test, *p* < 0.01). However, the changes in the mean enrichments between the lowest and highest regimes were small with 14% and 12% for wind speed and solar radiation, respectively.

### Variability of FDOM enrichments in the fjords

**Figure 4:**
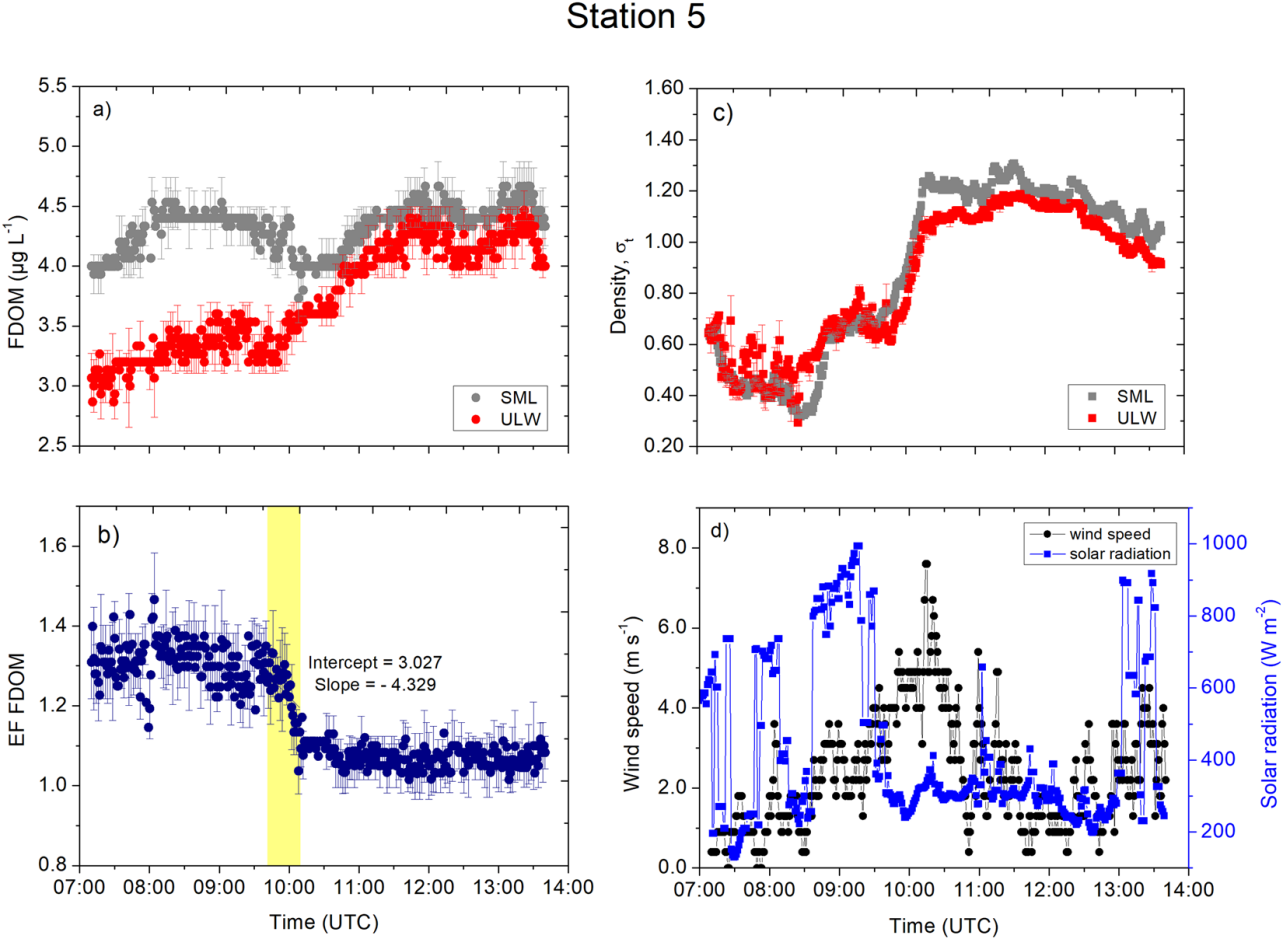
Measurement taken at Station 5. (**a**) The FDOM concentrations (in the SML and the ULW). (**b**) The enrichment factor of the FDOM. (**d**) The density (in the SML and the ULW). (**d**) The wind speed and the solar radiation were measured at 1 min intervals. Symbols represent a 1 min average. Error bars indicate ± standard deviation. EF: enrichment factor. FDOM: fluorescent-dissolved organic matter. SML: sea surface microlayer. ULW: underlying water. σ_t_: density at a given temperature and salinity minus 1000 kg m^-3^.

We performed high-resolution measurements (0.1 Hz) of the FDOM concentrations and other physical parameters (plotted as 1 min averages) in order to observe the processes controlling the enrichment factors of the FDOM in the SML. At the inner station of the Sogne Fjord (Station 5), we observed a consistent trend in the FDOM concentration in the SML (Fig. 4a); the concentration ranged narrowly between 4.0 µg L^−1^ and 4.5 µg L^−1^ (average: 4.3 ± 0.2 µg L^−1^, *n* = 2376). Meanwhile, the FDOM in the ULW approached with an increasing trend the concentrations of the SML, i.e., from 2.8 µg L^−1^ to 4.0 µg L^−1^ (average: 3.7 ± 0.4 µg L^−1^, *n* = 2376). The variance of FDOM concentrations in the SML and ULW were unequal (F-test, *p* < 0.0001) with a higher standard deviation in the ULW (Table S1). The enrichment factor of the FDOM was consistently enriched (enrichment factor >1) over the sampling period (Fig. 4b) but decreased close to the enrichment factor = 1.1 at 10:00 UTC, concurrent with the steepest increase in the concentration in the ULW, at a decreasing rate of 0.002 min^−1^. The similar densities of the SML and the ULW (Fig. 4c) showed an increasing trend over the sampling period and showed the water masses mixed until 10:00 UTC. Interestingly, a density anomaly is evident at the peak of wind speeds of approximately 7.0 m s^−1^ at 10:00 UTC. We also observed a similar trend in the high precision measurement of temperature (HPT) measured at approximately <2 cm and >15 cm, respectively (Fig. S2a).

**Figure 5:**
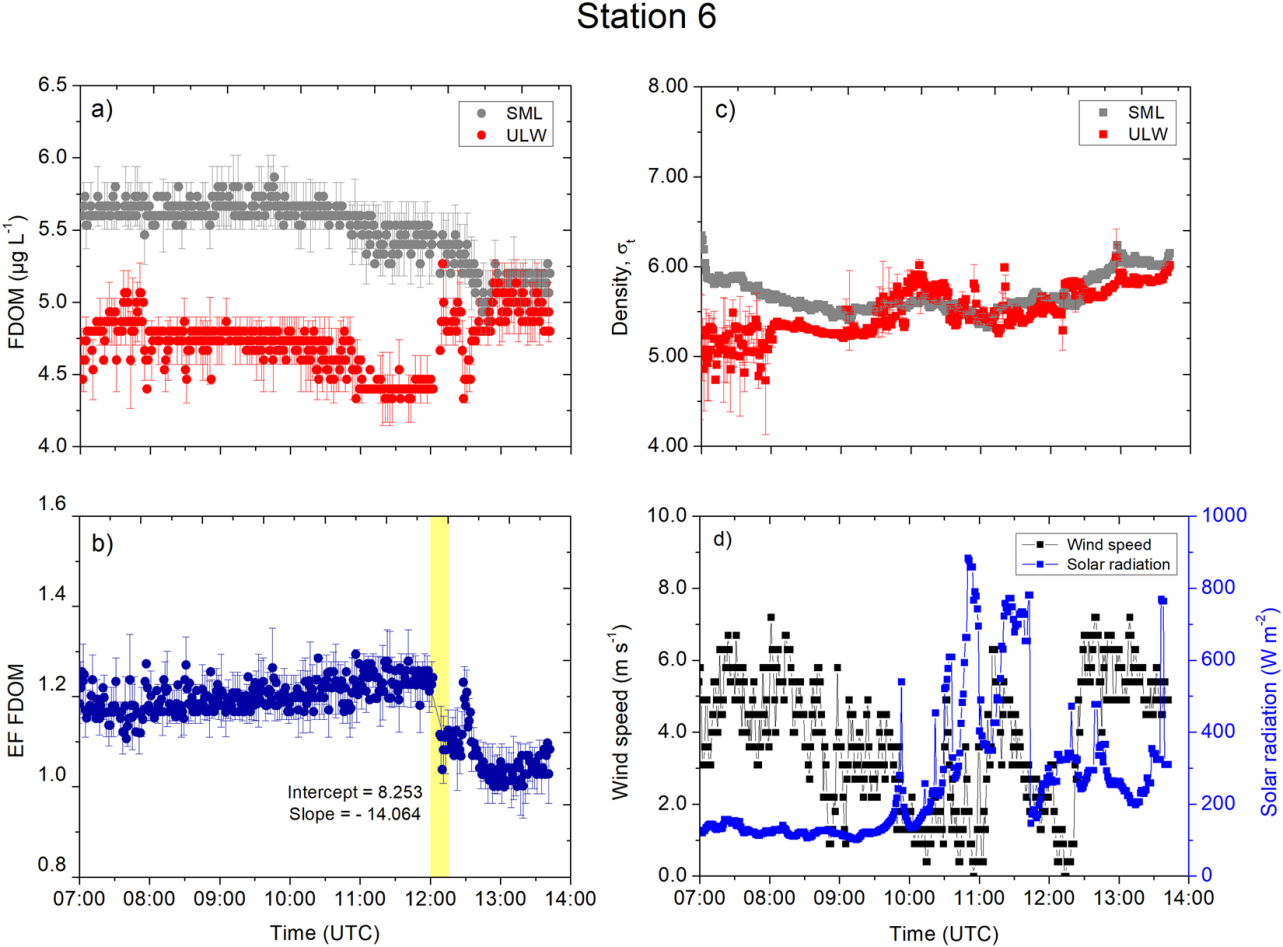
Measurement taken at Station 6. (**a**) The FDOM concentrations (in the SML and the ULW). (**b**) The enrichment factor of the FDOM. (**c**) The density (in the SML and the ULW). (**d**) The wind speed and the solar radiation were measured at 1 min intervals. Symbols represent a 1 min average. Error bars indicate ± standard deviation. EF: enrichment factor. FDOM: fluorescent-dissolved organic matter. SML: sea surface microlayer. ULW: underlying water. σ_t_: density at given temperature and salinity minus 1000 kg m^−3^.

Station 6, located 16 nautical miles south of Station 5, was also consistently enriched (enrichment factor >1) over the sampling period (Fig. 5). The FDOM concentration in the SML varied from 4.9 µg L^−1^ to 5.8 µg L^−1^ (average: 5.2 ± 0.2 µg L^−1^, *n* = 2453), and the FDOM in the ULW varied within a similar range from 4.3 µg L^−1^ to 5.3 µg L^−1^ (average: 4.9 ± 0.2 µg L^−1^, *n* = 2453). An F-test confirms equal variance of FDOM concentrations in the SML and ULW (*p* = 0.5062). Indeed, the FDOM concentrations in the SML decreased sharply at 12:00 UTC approaching a concentration of about 5.0 µg L^−1^ (Fig. 5a), and concurrent concentrations in the ULW increased similarly approaching the levels of the concentrations in the SML. Consequently, the enrichment factor was controlled from the SML and the ULW at the same time point and approached unity. The enrichment factor of the FDOM (Fig. 5b) decreased to about unity at 12:00 UTC at a rate of 0.018 min^−1^, i.e., 0.5 h before the wind speed increased to 7.6 m s^–1^ (Fig. 5d). The increased wind speed, the concurrent decrease in the FDOM concentrations in the ULW, and the remarkably constant concentrations in the SML caused a light trend of increasing enrichment factors. However, the wind was similarly strong between 07:00 and 08:00 UTC with a constant enrichment factor of 1.2 indicating that the wind did not always influence enrichment processes as researchers have often assumed^15^.

**Figure 6:**
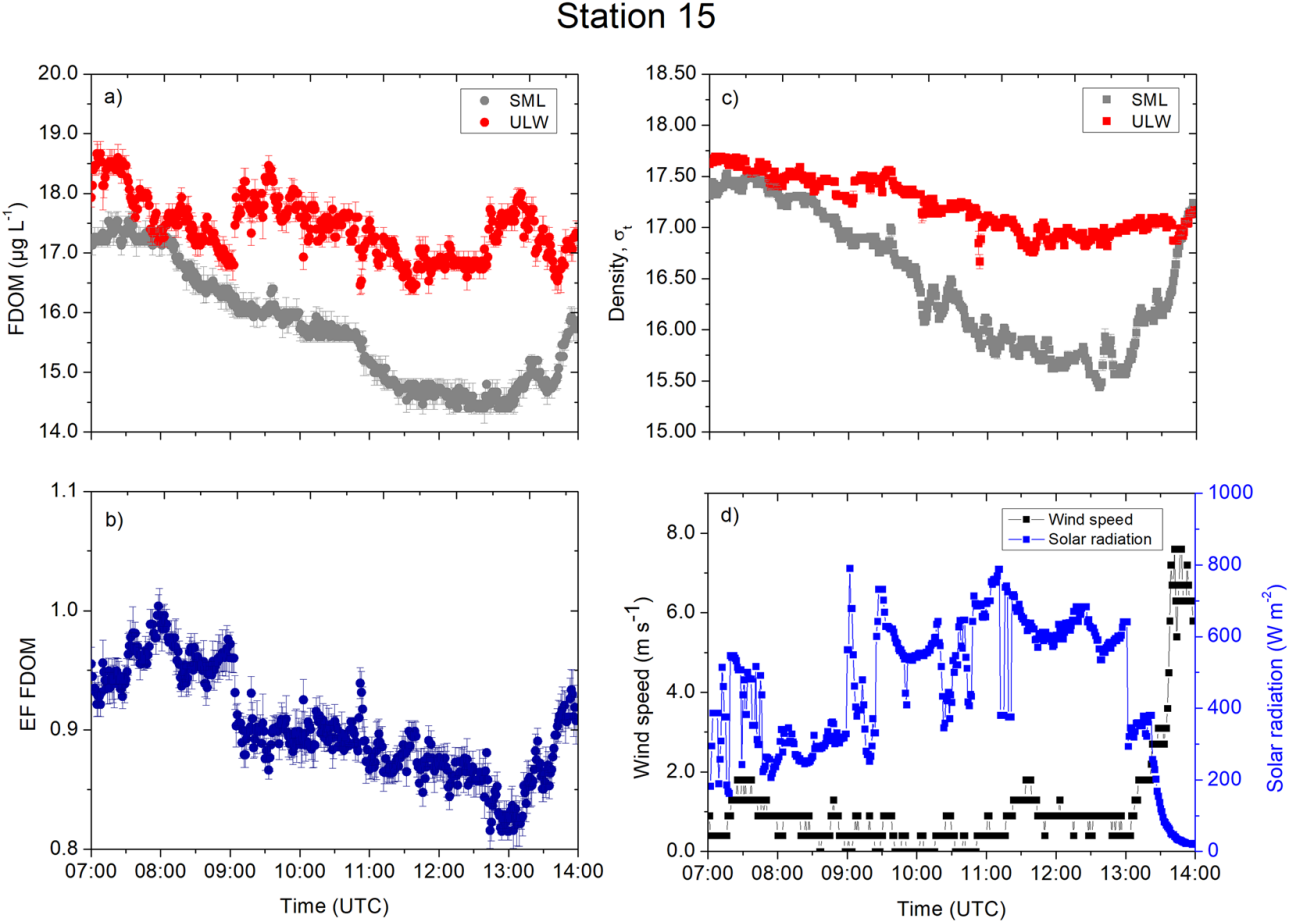
Measurement taken at Station 15. (**a**) The FDOM concentrations (in the SML and the ULW). (**b**) The enrichment factor of the FDOM. (**c**) The density (in the SML and the ULW). (**d**) The wind speed and the solar radiation were measured at 1 min intervals. Symbols represent a 1 min average. Error bars indicate ± standard deviation. EF: enrichment factor. FDOM: fluorescent-dissolved organic matter. SML: sea surface microlayer. ULW: underlying water. σ_t_: density at given temperature and salinity minus 1000 kg m^−3^.

Station 15 showed another distinct pattern of FDOM concentrations (Fig. 6) controlling the enrichment. Statistically significantly higher FDOM concentrations by a factor of at least three (Table S1) characterized the SML and the ULW compared to the other stations (Kruskal-Wallis with Dunn multiple test, *p* < 0.0001). Due to the proximity of Station 15 to the shoreline with potential land run-off, this station had fresh inputs of FDOM from terrestrial sources. The temporal FDOM concentrations in the ULW were more consistent (range: 16.4–18.8 µg L^−1^, average: 17.4 ± 0.5 µg L^−1^, *n* = 2507). Meanwhile, the FDOM concentration in the SML ranged from 14.0 to 17.6 µg L^−1^ (average: 15.7 ± 1.0 µg L^−1^, *n* = 2507) and showed a decreasing trend between 09:00 and 13:00 UTC. Although we measured higher FDOM concentrations in the SML and the ULW, the FDOM was consistently depleted (enrichment factor <1) over the sampling period (Fig. 6b). The average enrichment factor over the first 2 h (1.0 ± 0.02, *n* = 121) was statistically significantly higher (Mann-Whitney test, *p* > 0.0001) compared to the remaining sampling period, as the enrichment factor decreased to an average of 0.9 ± 0.03 (*n* = 296). Coincident with the decrease in the FDOM concentrations in the SML and the enrichment factor, the solar radiation increased from an average of 327 ± 96 W m^−2^ (*n* = 120) to 505 ± 197 W m^−2^ (*n* = 297; Fig. 6d). The increase in solar radiation (Fig. 6d) caused constant warming of the SML and the ULW (Fig. S4c), and the water masses of the SML and the ULW evolved similarly in terms of temperature and salinity (see the T-S diagram in Fig. 1). In addition, we observed increasing trends of FDOM concentrations between 13:30 UTC and 14:00 UTC, and the equal densities between the SML and the ULW (Fig. 6c) indicate complete mixing occurred due to increasing wind speeds to 7.5 m s^−1^ (Fig. 6d). At the same time, we visually observed rain near Station 15, which might have contributed to increasing the FDOM concentration due to land run-off. The SML received 50% more FDOM than the ULW within 30 min, e.g., 1.5 µg L^−1^ (SML) versus 1.0 µg L^−1^ (ULW) during the last hour of observation.

### Variability of FDOM enrichments in the open ocean

**Figure 7:**
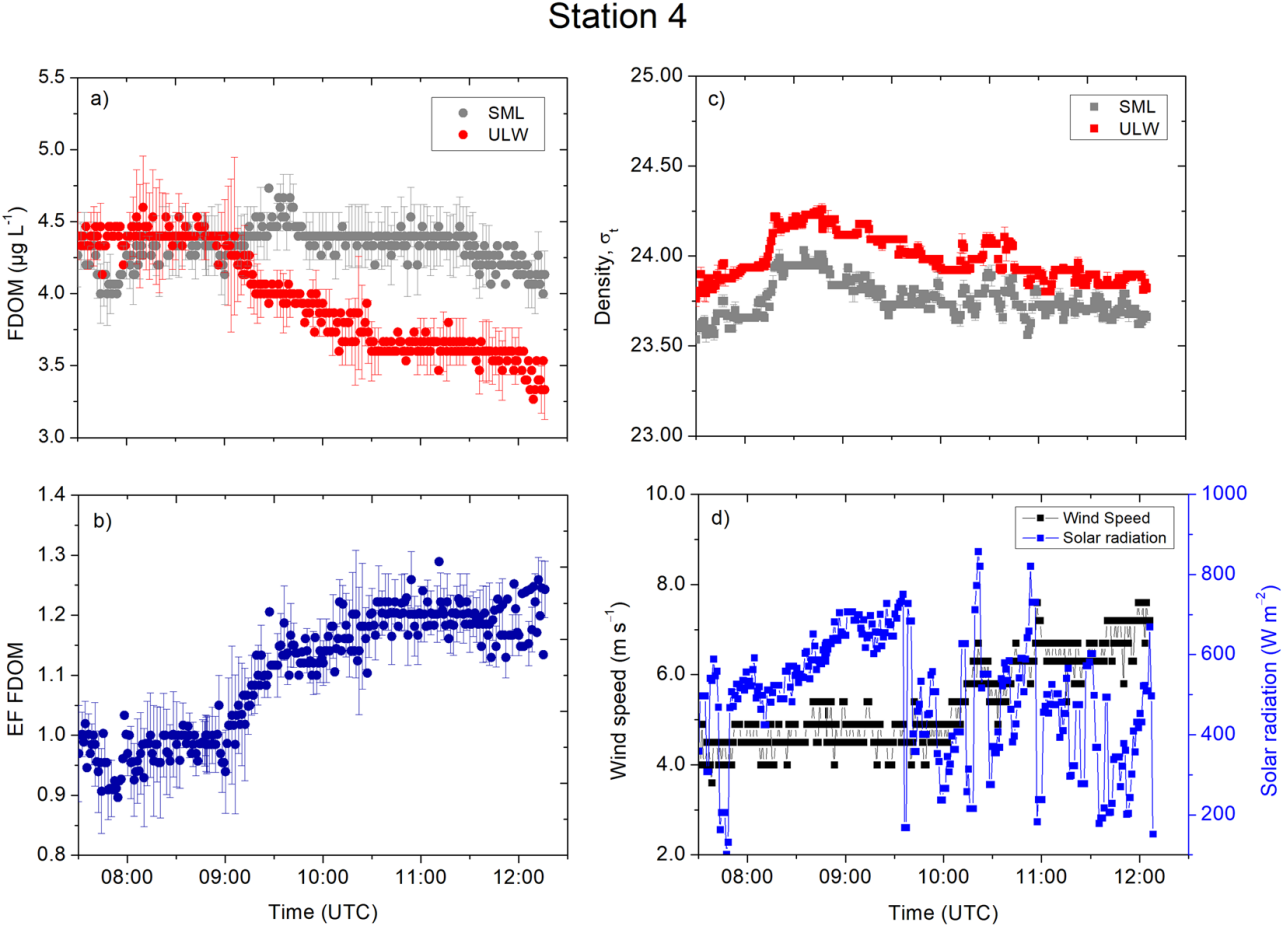
Measurement taken at Station 4. (**a**) The FDOM concentrations (in the SML and the ULW). (**b**) The enrichment factor of the FDOM. (**c**) The density (in the SML and the ULW). (**d**) The wind speed and the solar radiation were measured at 1 min intervals. Symbols represent a 1 min average. Error bars indicate ± standard deviation. EF: enrichment factor. FDOM: fluorescent-dissolved organic matter. SML: sea surface microlayer. ULW: underlying water. σ_t_: density at given temperature and salinity minus 1000 kg m^−3^.

**Figure 8:**
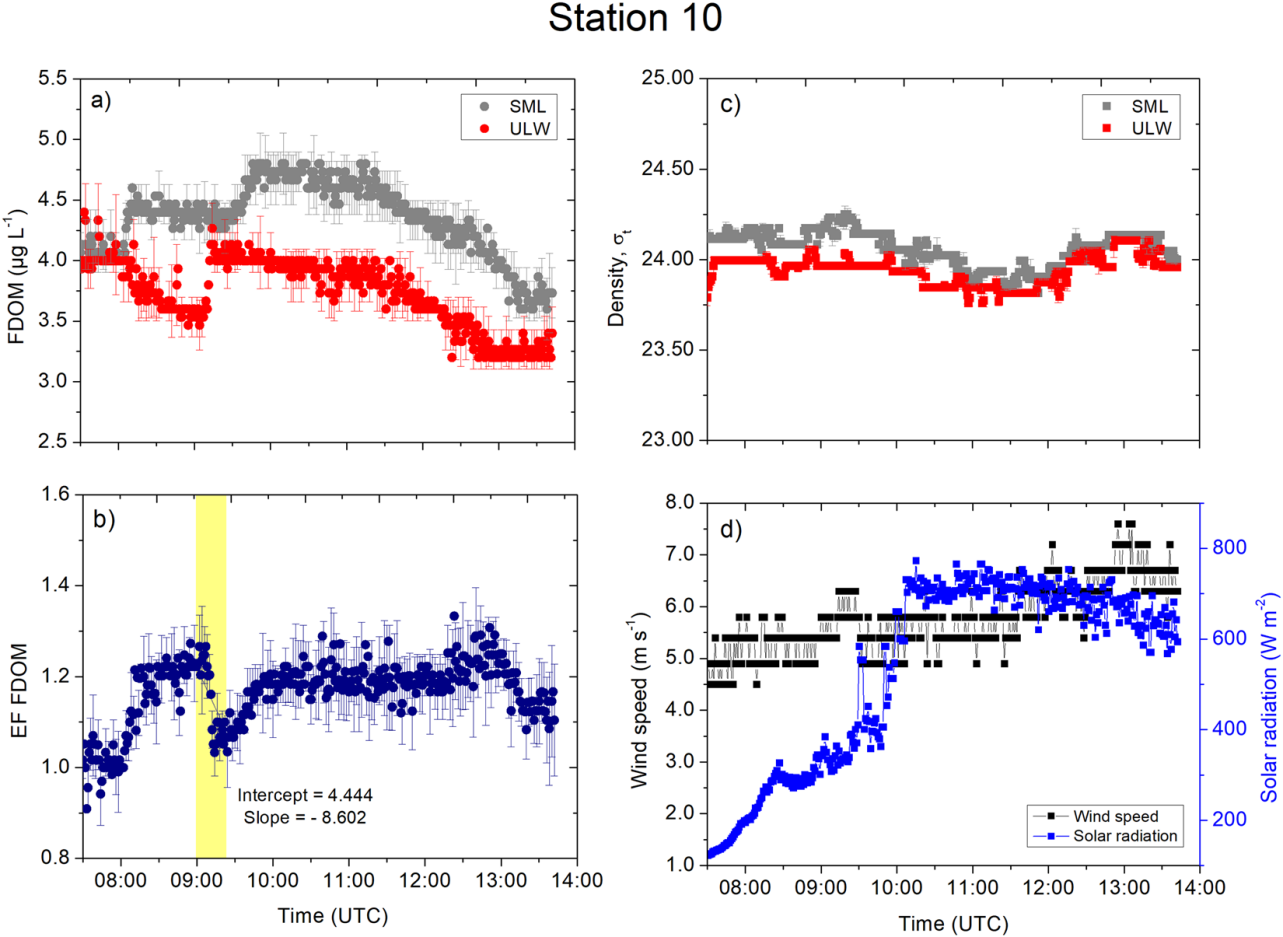
Measurement taken at Station 10. (**a**) The FDOM concentrations (in the SML and the ULW). (**b**) The enrichment factor of FDOM. (**c**) The density (in the SML and the ULW). (**d**) The wind speed and the solar radiation were measured at 1 min intervals. Symbols represent a 1 min average. Error bars indicate ± standard deviation. EF: enrichment factor. FDOM: fluorescent-dissolved organic matter. SML: sea surface microlayer. ULW: underlying water. σ_t_: density at given temperature and salinity minus 1000 kg m^−3^.

Processes in the ULW controlled the enrichment as we observed at the three stations located in the open North Atlantic. Station 4 (Fig. 7) showed a consistent FDOM concentration in the SML ranging between 4.0 µg L^−1^ and 4.7 µg L^−1^ (average: 4.3 ± 0.2 µg L^−1^, *n* = 1733; Table S1, Fig. 7a). The FDOM concentration in the ULW ranged between 3.3 µg L^−1^ and 4.6 µg L^−1^ (average: 4.0 ± 0.4 µg L^−1^,* n* = 1733). The variance in the concentrations were unequal (F-test, *p* < 0.0001), and higher in the ULW as indicated by the higher standard deviation. The enrichment factor of the FDOM ranged between 0.9 and 1.4 (average: 1.0 ± 0.1, *n* = 1733). The enrichment factor increased from 1.0 to 1.2 at 09:00 to 10:30 UTC (Fig. 7b) because the FDOM concentration in the ULW decreased by 22%. Located in the same oceanic regime, the observations at Station 10 showed a more distinct decrease in the FDOM concentration in the SML (Fig. 8a; average: 4.3 ± 0.3 µg L^−1^, *n* = 2084). The FDOM concentration in the ULW ranged between 3.2 µg L^−1^ and 4.8 µg L^−1^ (average: 3.7 ± 0.3 µg L^−1^, *n* = 2084). The enrichment factor of the FDOM concentration increased from unity to 1.3; i.e., approximately by 30% between 08:00 and 09:00 UTC (Fig. 8b) due to a 0.4 µg L^−1^ increase in the concentration in the SML and a concurrent 1.5 µg L^−1^ decrease in the concentration in the ULW. The changes in the enrichment factor continued with the increasing concentrations in the ULW at 09:00 UTC; e.g., the enrichment factor dropped to 1.1 at a rate of 0.009 min^−1^ (Fig. 8b). The subsequent increase in the concentration in the SML at 09:30 UTC caused the enrichment factor to rise and remain at 1.2 despite moderate wind speeds of 6.0 to 7.0 m s^−1^ (Fig. 8d). The densities of the SML and the ULW (Fig. 8c) showed a similar trend, and they were less distinct from each other (see the T-S diagram in Fig. 1), suggesting we likely conducted the measurements in the same water masses due to wind-driven mixing. However, the enrichment of potentially hydrophobic FDOM in the SML was consistent throughout the sampling period. Interestingly, the high precision temperature data show that the surface water (<2 cm) was warmer than the underlying water (>15 cm; Fig. S2c), and we did not observe a direct relation with solar radiation (Fig. 8d).

**Figure 9:**
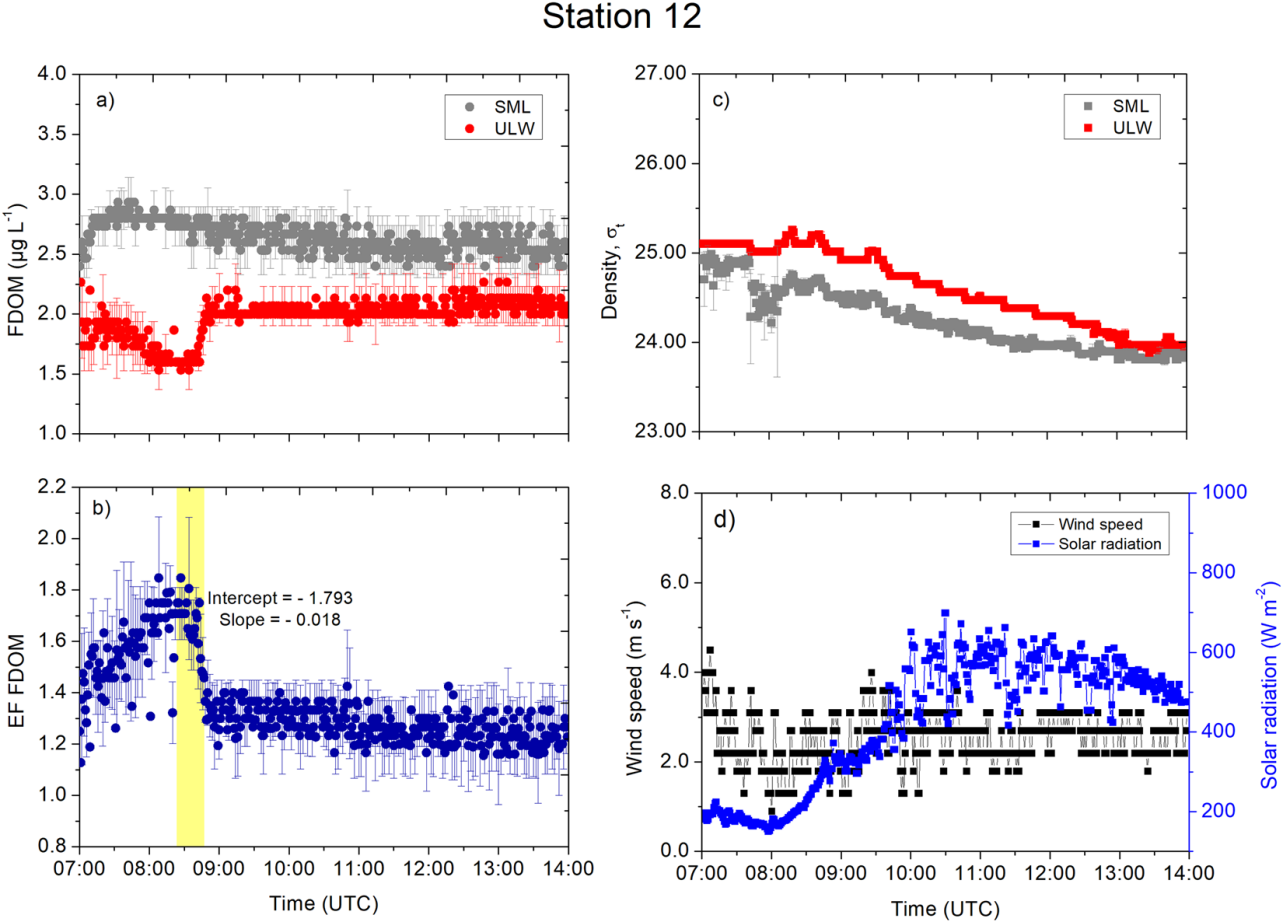
Measurement taken at Station 12. (**a**) The FDOM concentrations (in the SML and the ULW). (**b**) The enrichment factor of the FDOM. (**c**) The density (in the SML and the ULW). (**d**) The wind speed and the solar radiation were measured at 1 min intervals. Symbols represent a 1 min average. Error bars indicate ± standard deviation. EF: enrichment factor. FDOM: fluorescent-dissolved organic matter. SML: sea surface microlayer. ULW: underlying water. σ_t_: density at given temperature and salinity minus 1000 kg m^−3^.

Station 12, located approximately 107 nautical miles north from Station 10, had the lowest FDOM concentration in the SML (range: 2.0–3.2 µg L^−1^) and the ULW (range: 1.6–2.4 µg L^−1^; Fig. 9) probably due to the station’s location in the open ocean. In contrast to Station 10, the low wind condition at Station 12 (Fig. 9d) led to stable stratification (see the T-S diagram in Fig. 1). As a consequence, the pattern of the FDOM concentration in the SML was less variable compared to the concentrations in the ULW. The decrease in the FDOM in the ULW between 07:00 and 08:30 UTC (Fig. 9a) caused the enrichment factor to increase by 63%, and the enrichment factor reached a maximum of 1.8 at 08:30 UTC (Fig. 9b). However, the enrichment factor decreased sharply after 08:30 UTC at a decreasing rate of 0.006 min^−1^ due to a distinct increase in the concentrations in the ULW by 33%. Solar radiation increased from 150 W m^−2^ (08:00 UTC) to 700 W m^−2^ (10:30 UTC; Fig. 9d) without any effects on the FDOM in the SML (Fig. 9a).

## Discussion

In the present study, we showed that the magnitude of enrichment in the SML is not solely governed by processes in the SML itself, as is often assumed^15,23^. Even though we observed slightly lower enrichments with increasing wind speed and solar radiation, sudden changes in the concentration in the ULW can be sufficiently high to control the enrichment of FDOM in the SML. For decades, researchers have stated that the ULW is a major source of organic material for the SML^15^. However, our finding that changes in the bulk concentrations and the mixing of two water masses are sufficient to control the enrichment process is novel and alters the perspective on the formation of the SML and the interpretation of enrichment factors.

Generally, this study showed that FDOM is enriched in the SML, and the enrichment factors are comparable with other observations in the western North Atlantic^5^, Baltic Sea^24,25^ and North Sea^25^ with the enrichment factor not exceeding 1.7. These observations include sampling in upwelling regions, fronts and during rain events^5,24,25^. We conclude that FDOM enrichment is a general feature of the SML despite different sampling regions and potential sources. This finding is remarkable as complex mechanisms, including wind forces, solar and ultraviolet (UV) radiations, and *in situ* production^24,26,27^, drive the enrichment of FDOM in the SML.

The inner Sogne Fjord (Station 5, Fig. 4) is influenced by the proximity to the Jostedalsbreen glacier. It has been shown that FDOM in glacial melt water is associated to proteinaceous material, and to a lesser extent to aromatic compounds^28^. However, our FDOM measurements (excitation wavelength: 370 nm, emission wavelength: 460 nm) is sensitive to highly aromatic humic substances^29^ and explains the relatively low concentrations of FDOM at Station 5 and 6. Nevertheless, the increasing FDOM concentration in the ULW coincident with increasing wind speed (Fig. 4d) with probable complex hydrodynamic mixing of deeper fjord water with glacial melt water. The wind-driven mixing of with deeper water caused the ULW to increase in FDOM concentrations ULW and consequently contributed to decreasing enrichment factors at 10:00 UTC (Fig. 4b). Besides wind mixing, tidal mixing may affect the FDOM enrichment process. However tidal mixing typically occurs in time scale of hours^30^, and not within minutes as observed in this study (Fig. 4b). Due to photochemical bleaching^31^ it is expected that the maximum of FDOM concentrations is located below 1 m depth (ULW depth in this study), while it is assumed that recalcitrant FDOM becomes enriched in the SML as further discussed below. Located in the middle fjord and close to other fjord branches (Fig. 2), ULW at Station 6 is probably well mixed by hydrodynamics, but with an overlying cooler SML (see the T-S diagram in Fig. 1). A recent study on the bacteria community in the Sogne Fjord^32^ discussed that locations in the center of the fjord could be an active mixing zone with inflowing bacteria populations from the southern side and outflowing populations to the northern side. In fjord systems, wind forcing from different directions potentially interacts in an antagonistic pattern and causes mixing^21^. Upwelling of underlying water masses compensate wind-forced mixing processes by Ekman transport counteracting the sinking of surface water^33^. This process could explain the mixing of the ULW with SML between 12:00 and 14:00 UTC (Fig. 5a) with sudden changes in wind speeds from 0 to 7.0 m s^−1^ within 10 min (Fig. 5d). However, as wind waves in fjords are limited by fetch, such wind-driven mixing of the SML and ULW depends not only on the wind speed, but also on the wind direction and topography of the fjord. The variability of internal tides in the fjord is influenced by high-wave energy and wind-driven exchange processes between offshore and inshore fjord waters^34^. Such processes may have contributed to our observations, but to our knowledge the effect of internal tides on the SML has not been investigated. Moreover, the estuarine circulation and tidal motions in the Sogne fjord significantly affects the horizontal transport of allochthonous and autochthonous sources of DOM within the surface and intermediate water layers^32^, but without reference to the SML.

It is clear from our observation that fresh inputs of FDOM occurred in the Trondheim Fjord (Station 15), probably due land run-off during a non-local rain event. The moderate average solar radiation (453 ± 192 W m^−2^, *n* = 417, Table S1) at Station 15 seemed to be sufficient to cause depletion of fresh FDOM in the SML with potentially more rapid photochemical degradation in the SML than in the ULW^3^. Researchers reported that terrigenous DOM is more reactive to biological and photochemical degradation^35^, explaining the constant enrichment at the stations in the open Atlantic despite similar or higher solar radiations. Photochemical degradation in the SML as observed in this study is an important process altering the optical properties of DOM^36^. In addition, non-local rain from nearby land (visual observation) and increasing wind speed up to 7.6 m s^−1^ consequently increased the enrichment factor by 7% within 30 min. It included cooling of surface water (depth 2 cm) by 1.5 °C (Fig. S2e), similar to earlier observations^37^. Fresh water inflow from land supplies terrestrial-derived DOM directly into the fjord, and therefore increased the FDOM concentration. However, the increasing value was smaller compared to observations during a local rain event^25^, when the enrichment factor increased up to two-fold within approximately 15 min probably due to the local precipitation as additional source of FDOM^38^.

At the open ocean stations, changes in the FDOM concentrations in the SML and the ULW were not in line with the time series trends of the densities and their anomalies. For example, stable stratification at Station 4 (Fig. 7c) and Station 12 (Fig. 9c) separated the SML from underlying processes and inhibited sinking of the SML water with the consequence of less variable FDOM concentrations in the SML. The mixing process near the sea surface is very complex and dynamic. For example, Langmuir turbulence can penetrate turbulent kinetic energy to deeper waters, changing mixed layer depth and therefore indirectly affecting enrichment processes in the SML. However, we did not observe white parallel streaks as indicator for the presence of Langmuir circulation. Indeed, we suggest that the catamaran crossed different underlying water masses, e.g. at Station 12 at 08:00 UTC (Fig. 9c), due to the single sudden change in FDOM concentrations followed by constant concentrations over several hours. For Station 10, the increase in the enrichment factor by up to 30% corresponded to a decrease in the concentrations in the ULW between 08:00 and 09:00 UTC. Researcher previously reported similar changes^5^, where the enrichment factor of the FDOM changed from approximately 1.4 to 1.0 within 1 h. Due to evaporative cooling, the SML is typically tenth’s degree cooler than the ULW^37^ driving convective mixing. Warming of the upper surface layer (Fig. S2) in the morning can influence convective mixing depending on meteorological conditions. For example, at Station 10 (Fig. S2c), the initial cooler layer at >15 cm warms up approaching the temperature across the <2 cm layer, and therefore, slowing down convective mixing. At Station 15, peaks in solar radiation (11:00 UTC to 12:30 UTC, Fig. 6d) caused the <2 cm layer to warm up (Fig. S2e) and therefore convection mixing may intensify, which could not be quantified in this study. Similar, at Station 6 solar radiation peaked at 11:00 UTC (Fig. 5d), which caused the <2 cm layer to warm up (Fig. S2b). Stations 5 (Fig. S2a) and 12 (Fig. S2d) shows similar trends in the temperature at <2 cm and >15 cm, i.e., likely small influences on enrichment processes by convective mixing.

The wind speed remains to force enrichment processes. For example, at Station 4, increasing wind speed caused a partial mixing of ULW with SML as shown by decreasing density anomalies, i.e. weakening the stratification. Above the threshold value for breaking waves (i.e., 2.5 m s^−1^)^39^, higher wind speeds can promote partial mixing of the SML and the ULW, i.e. Station 4 at 10:00 UTC (Fig. 7d) and Station 10 at 12:00 UTC (Fig. 8d). In addition, higher wind speeds enhanced bubble formation through air entrainment by breaking waves, and scavenged DOM from the ULW to the SML^40^. For example^41^, providing evidence that the scavenging process by bubbles from the ULW is a major source for the enrichment of dissolved amino acids and bacteria in the SML. The similar enrichment factor patterns in all open ocean stations were not affected by solar radiation, supporting evidence that FDOM in the open ocean is more recalcitrant^42^. The long residence time of the surface water and consequently prolonged exposure to solar radiation cause depletion of labile DOM, including FDOM, in the open ocean^43^. This process is enhanced in the SML as observed at the coastal Station 15 with fresh FDOM, and enriched FDOM in the open ocean is probably very recalcitrant in the SML. Enriched and recalcitrant FDOM can potentially behave as a protective substance for phytoplankton in the SML against the strongest solar radiation throughout the water column as suggested previously^27^.

Overall, we found that the FDOM concentrations in the SML can be less variable compared to the ULW despite the layer’s position at the air–sea interface which is exposed to extreme physical and meteorological forcing. We showed that changes in the concentrations in the ULW are distinct and sufficient to control the enrichment of FDOM in the SML in different oceanic regimes. Regardless of the multiple processes controlling the enrichment of FDOM in the SML (e.g., wind speed, surface renewal and photo degradation^24^) we conclude that the underlying water masses and mixing processes require more attention in future studies of enrichment processes in the SML.

## Materials and Methods

We collected the data for this study during the HE491 cruise on the R/V Heincke from July 8 to July 28, 2017. Stations 5 and 6 were located in the inner and middle parts of the Sogne Fjord (Norway), respectively. Stations 4, 10, and 12 were located in the North Atlantic Ocean (Fig. 2) at least 80 nautical miles offshore. Station 15, located in the inner Trondheim Fjord (Norway), presents an area with fresh inputs of terrestrial FDOM due to the geographic features of this fjord and precipitation during our observations. At every station, we deployed the remote-controlled catamaran Sea Surface Scanner^25^ to collect *in situ* data from the SML and the ULW (1 m depth). Briefly, we collected continuously in the SML with six rotating glass disks (diameter 60 cm, thickness 0.8 cm) mounted between the hulls of Sea Surface Scanner^25^. We immersed the glass disks to a water depth of 15 cm and rotated them at 7 rotations per minute. The SML adhered to the disks by the phenomenon of surface tension while the disks rotated through it. We scraped off the collected SML with a set of polycarbonate wipers mounted between the glass disks. By gravity, the SML flowed in a collection vessel from which the SML was pumped through a flow cells of an FDOM sensor (MicroFlu-CDOM, Trios GmbH, Germany; excitation wavelength: 370 nm, emission wavelength: 460 nm) and a conductivity and temperature cell (Model: MU6010H, VWR, Germany). Simultaneously, water from a depth of 1 m (i.e., the ULW) is pumped through a second set of flow cells. We measured the FDOM, conductivity, and temperature in the SML and the ULW at the 0.1 Hz frequency. We recorded the raw fluorescent readings with a dual-channel data logger (Track-It, Monarch Instrument, USA) and transformed them into FDOM concentrations (µg L^−1^) based on the factory calibration scaling factor^24^. We blank corrected the values (average blank: 2.2 ± 0.5 µg L^−1^, *n* = 10) using ultra-pure water before every deployment. We calculated the enrichment factors of the FDOM as EF_FDOM_ = SML_FDOM_/ULW_FDOM_. We derived the salinity from the conductivity data and computed the density (sigma-t), using the equation of state for seawater^44^. Sigma-t is defined as density at a given temperature and salinity minus 1000 kg m^-3^. We calculated the salinity, temperature, and density anomalies as ΔX = X_SML_ − X_1m_; X represents the salinity, temperature, or density. In addition, we measured the temperature with high accuracy (±0.015 °C; P795, Dostman Electronic GmbH, Germany) *in situ* at <2 cm and >15 cm depths at a frequency of 0.1 Hz. We used an active fluorometer PhytoFlash (Turner Designs, Sunnyvale, CA, USA) to evaluate the physiological status of autotrophic organisms in the ULW. The PhytoFlash detection system uses three low-intensity LEDs to monitor minimum florescence (F_o_), maximum fluorescence (F_m_), and variable fluorescence (F_v_: F_m_ − F_o_). The quantum yield (F_v_/F_m_) determines how well phytoplankton can assimilate light for photosynthesis and therefore, their physiological status. Before and after the cruise, we calibrated the PhytoFlash with reference measurements to a benchtop fluorometer according to the supplier’s guidelines. A weather station (Model: VantagePro 2, Davis Instrument, USA) mounted on the mast of the Sea Surface Scanner at a height of 3 m recorded meteorological parameters, including wind speed and direction, air temperature, humidity, rain rate, and solar and UV radiation. We have published additional details of the sampling technique and *in situ* data measurement elsewhere^25^.

## Electronic supplementary material

Supplementary information

## Data Availability

Data used in this study are available from the authors upon request.
