# Supplementary material for: High-resolution observations on enrichment processes in the sea-surface microlayer
Source: Sci Rep. 2018 Sep 3;8:13122. doi: 10.1038/s41598-018-31465-8 (PMC6120901; doi:10.1038/s41598-018-31465-8)
Supplement: Supplementary file 1 — Supplementary information [file 41598_2018_31465_MOESM1_ESM.docx]

Supplementary

High-resolution observations on enrichment processes in the sea-surface microlayer

Nur Ili Hamizah Mustaffa^*^, Thomas H. Badewien, Mariana Ribas-Ribas & Oliver Wurl

*Corresponding author: N.I.H.M. (Email: [nur.ili.hamizah.mustaffa@uol.de](mailto:nur.ili.hamizah.mustaffa@uol.de))

Supplementary Table

**Table S1.** Biochemical and meteorological parameters measured at each station. Values reported as average ± standard deviation

|  | Stations | | | | | |
| --- | --- | --- | --- | --- | --- | --- |
|  | Station 4 | Station 5 | Station 6 | Station 10 | Station 12 | Station 15 |
| Date | 11-July-2017 | 12-July-17 | 13-July-17 | 19-July-17 | 21-July-17 | 25-July-17 |
| Location | North Atlantic | Sogne fjord | Sogne fjord | North Atlantic | North Atlantic | Trondheim fjord |
| Station feature | Open ocean | Inner fjord | middle fjord | Open ocean | Open ocean | Inner fjord |
| Latitude (°N) | 60.82859 | 61.35759 | 61.08901 | 62.59515 | 63.91813 | 63.52424 |
| Longitude (°E) | 3.93269 | 7.36705 | 6.99928 | 4.38205 | 7.02626 | 10.40883 |
| FDOM, SML | 4.3 ± 0.2 | 4.3 ± 0.2 | 5.2 ± 0.2 | 4.3 ± 0.3 | 2.6 ± 0.2 | 15.7 ± 1.0 |
| (µg L^–1^) | (*n* = 1733) | (*n* = 2376) | (*n* = 2453) | (*n* = 2084) | (*n* = 2489) | (*n* = 2507) |
| FDOM, ULW | 4.0 ± 0.4 | 3.7 ± 0.4 | 4.9 ± 0.2 | 3.7 ± 0.3 | 2.0 ± 0.2 | 17.4 ± 0.5 |
| (µg L^–1^) | (*n* = 1733) | (*n* = 2376) | (*n* = 2453) | (*n* = 2084) | (*n* = 2489) | (*n* = 2507) |
| EF FDOM | 1.0 ± 0.1 | 1.2 ± 0.1 | 1.2 ± 0.1 | 1.2 ± 0.1 | 1.3 ± 0.2 | 0.9 ± 0.04 |
|  | (*n* = 1733) | (*n* = 2376) | (*n* = 2453) | (*n* = 2084) | (*n* = 2489) | (*n* = 2507) |
| Photosynthetic Yield | NA | NA | NA | 0.2 ± 0.1 | 0.3 ± 0.1 | NA |
| (SML) |  |  |  | (*n* = 387) | (*n* = 492) |  |
| Photosynthetic Yield | 0.3 ± 0.2 | 0.2 ± 0.1 | 0.2 ± 0.1 | 0.2 ± 0.1 | 0.4 ± 0.2 | 0.3 ± 0.1 |
| (ULW) | (*n* = 1733) | (*n* = 2376) | (*n* = 2453) | (*n* = 1697) | (*n* = 1990) | (*n* = 2507) |
| Salinity, SML | 31.89 ± 0.12 | 2.33 ± 0.55 | 8.87 ± 0.87 | 32..17 ± 0.11 | 32.60 ± 0.36 | 23.63 ± 0.55 |
|  | (*n* = 1733) | (*n* = 2373) | (*n* = 2460) | (*n* = 2084) | (*n* = 2489) | (*n* = 2507) |
| Salinity, ULW | 32.23 ± 0.14 | 2.34 ± 0.48 | 8.43 ± 0.36 | 32.06 ± 0.17 | 33.10 ± 0.44 | 24.08 ± 0.34 |
|  | (*n* = 1733) | (*n* = 2373) | (*n* = 835) | (*n* = 2084) | (*n* = 2489) | (*n* = 2507) |
| Temperature, SML (°C) | 14.10 ± 0.17 | 14.64 ± 0.80 | 14.69 ± 0.29 | 13.64 ± 0.21 | 14.32 ± 0.41 | 17.73 ± 0.83 |
|  | (*n* = 1733) | (*n* = 2373) | (*n* = 835) | (*n* = 2084) | (*n* = 2489) | (*n* = 2507) |
| Temperature, ULW (°C) | 14.24 ± 0.20 | 14.70 ± 0.82 | 15.14 ± 0.21 | 13.83 ± 0.16 | 14.49 ± 0.43 | 17.51 ± 0.73 |
|  | (*n* = 1733) | (*n* = 2373) | (*n* = 835) | (*n* = 2084) | (*n* = 2489) | (*n* = 2507) |
| pH, SML | 8.12 ± 0.01 | 7.35 ± 0.12 | 8.06 ± 0.01 | 8.17 ± 0.01 | 8.18 ± 0.003 | 8.16 ± 0.01 |
|  | (*n* = 1733) | (*n* = 2373) | (*n* = 2460) | (*n* = 2084) | (*n* = 2489) | (*n* = 2507) |
| pH, ULW | 8.14 ± 0.01 | 7.59 ± 0.14 | 8.17 ± 0.01 | 8.20 ± 0.004 | 8.16 ± 0.004 | 8.21 ± 0.01 |
|  | (*n* = 1733) | (*n* = 2373) | (*n* = 835) | (*n* = 2084) | (*n* = 2489) | (*n* = 2507) |
| HPT, 2 cm (°C) | NA | 14.630 ± 0.799 | 14.982 ± 0.086 | 13.394 ± 0.129 | 13.809 ± 0.246 | 17.733 ± 0.833 |
|  |  | (*n* = 2373) | (*n* = 2460) | (*n* = 2084) | (*n* = 2489) | (*n* = 2507) |
| HPT, 15 cm (°C) | NA | 14.694 ± 0.816 | 15.039 ± 0.057 | 12.422 ± 0.875 | 13.818 ± 0.254 | 17.530 ± 0.730 |
|  |  | (*n* = 2373) | (*n* = 2460) | (*n* = 2084) | (*n* = 2489) | (*n* = 2507) |
| wind speed (m s^-1^) | 5.4 ± 1.0 | 2.3 ± 1.4 | 3.8 ± 1.9 | 5.8 ± 0.7 | 2.5 ± 0.6 | 1.1 ± 1.4 |
|  | (*n* = 287) | (*n* = 394) | (*n* = 414) | (*n* = 349) | (*n* = 410) | (*n* = 417) |
| Solar radiation (W m^–2^) | 498 ± 152 | 436 ± 221 | 268 ± 192 | 503 ± 226 | 416 ± 165 | 453 ± 192 |
|  | (*n* = 287) | (*n* = 394) | (*n* = 414) | (*n* = 349) | (*n* = 410) | (*n* = 417) |
| UV Index | 3.0 ± 0.7 | 2.6 ± 0.8 | 2.0 ± 1.2 | 3.4 ± 1.3 | 3.3 ± 1.0 | 3.4 ± 1.0 |
|  | (*n* = 278) | (*n* = 394) | (*n* = 414) | (*n* = 349) | (*n* = 367) | (*n* = 367) |
| NA - Not Available |  |  |  |  |  |  |

Supplementary figures


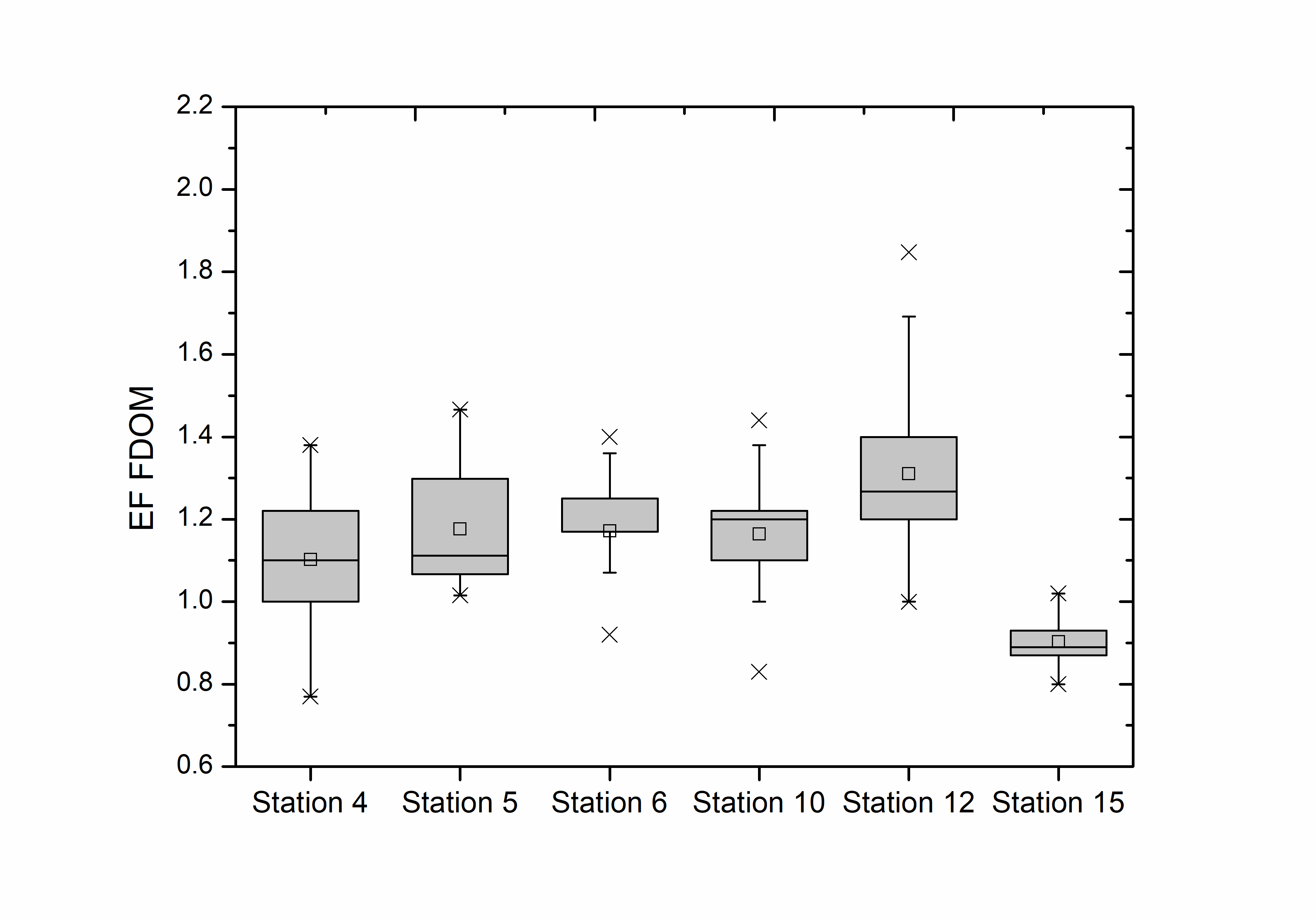


**Figure S1.** Box plot of enrichment factor (EF) of FDOM at every stations. Error bars shows median 25% and 75%, respectively. Line represent median 50%, unfilled square box represents mean values and cross symbols represent maximum and minimum values, respectively.


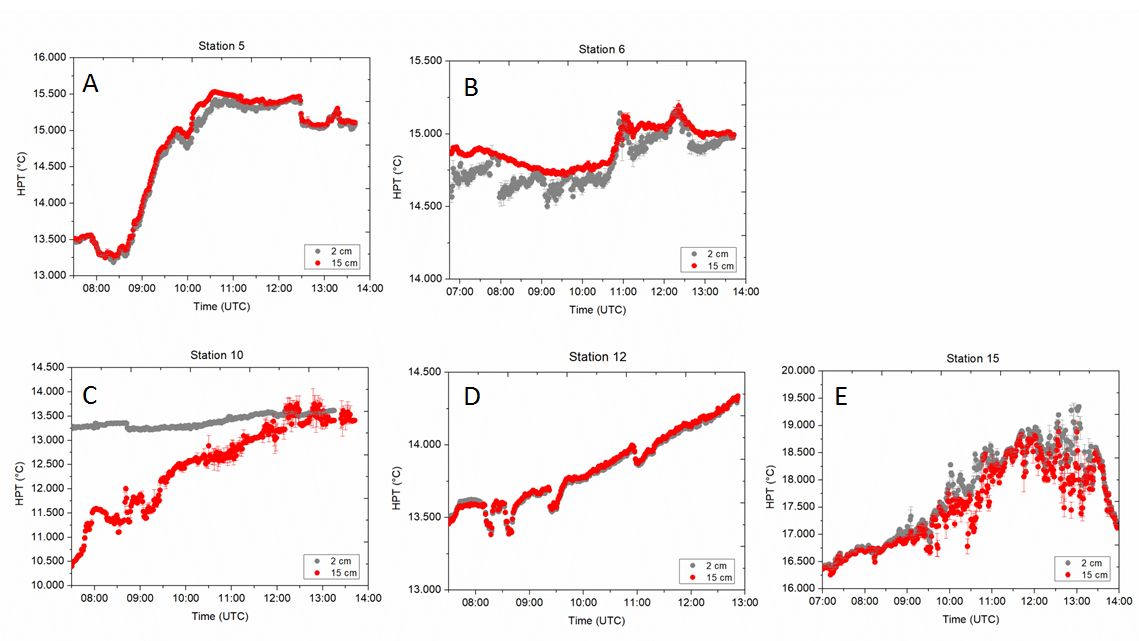


**Figure S2**. High precision temperature (HPT) measured at depth < 2cm and >15 cm. Symbols represent one-minute average, error bars indicates ± standard deviation.





**Figure S3**. Salinity data (in PSU) measured in the SML and ULW at every station. Symbols represent one-minute average, error bars indicates ± standard deviation.





**Figure S4**. Temperature measured in the SML and ULW at every station. Symbol represent one-minute average, error bars indicates ± standard deviation.
